# Prevalence of Asymptomatic Bacteriuria and Antibiotic Susceptibility Patterns of Bacterial Isolates among Cancer Patients and Healthy Blood Donors at the University of Gondar Specialized Hospital

**DOI:** 10.1155/2020/3091564

**Published:** 2020-04-16

**Authors:** Abiye Tigabu, Worku Ferede, Gizeaddis Belay, Baye Gelaw

**Affiliations:** Department of Medical Microbiology, School of Biomedical and Laboratory Sciences, University of Gondar, Gondar, Ethiopia

## Abstract

**Background:**

Urinary tract infections are the common types of infections in the community and health care settings. Despite the widespread availability of antibiotics, urinary tract infection remains a worldwide therapeutic problem. It is a continuous and significant problem in cancer patients.

**Methods:**

A hospital-based comparative cross-sectional study was conducted on 240 study participants from January to June 2019. Sociodemographic data were collected by a predesigned questionnaire and midstream urine samples collected using simple random sampling technique by using clean, sterile plastic cups and then inoculated onto CLED agar plates and incubated at 37°C for 24 hours. Urine culture was considered significant bacteriuria when colony forming units ≥10^5^/mL of voided urine and a single pure colony suspended in nutrient broth and then subcultured onto a blood agar plate and MacConkey agar plate, incubated at 37°C for 24 hours for identification. Identification was done by using standard microbiological methods. Modified Kirby–Bauer disk diffusion technique was applied for antimicrobial susceptibility testing in accordance with CLSI 2018 criteria. Data were entered, cleared, and checked using Epi Info version 7 and exported to SPSS version 20 for analysis. The results were displayed using tables and figures. *p* value <0.05 at 95% CI was considered as statistically significant.

**Results:**

The overall prevalence of asymptomatic bacteriuria in cancer patients was 23.3% while 6.7% in apparently healthy blood donors. *E*. *coli* (32.1%) was the commonest isolated uropathogenic bacteria followed by *Klebsiella* species (25.0%), *S*. *aureus* (21.4%), *Enterococcus* species (10.7%), *Serratia* species (7.1%), and *Enterobacter aerogenes* (3.6%) in cancer patients. In apparently healthy blood donors, *E*. *coli*, *Klebsiella* species, and *S. aureus* were isolated from 75%, 12.5%, and 12.5%, respectively. Most Gram-negative bacteria were more sensitive to ceftazidime, cefoxitin, nalidixic acid, nitrofurantoin, norfloxacin, ciprofloxacin, and tobramycin, whereas highly resistant to ampicillin, penicillin, tetracycline, and ceftazidime. *S*. *aureus* isolates were 100% susceptible to nitrofurantoin.

**Conclusions:**

This study showed a high prevalence of asymptomatic bacteriuria among cancer patients (23.3%) compared to apparently healthy blood donors (6.7%). *E*. *coli* was isolated predominately. Nitrofurantoin and ciprofloxacin should be used to treat asymptomatic bacteriuria in the study area.

## 1. Background

Cancer is the second cause of death next to cardiovascular diseases worldwide [1]. The most common location and highest mortality rate belong to pulmonary cancer in men and breast cancer in women. The International Agency for Research on Cancer (IARC) reported the most common cancers in both sexes are pulmonary, breast, colorectal, prostate, and gastric cancers [1, 2]. Infection is a continuous and significant problem in cancer patients due to many factors that increase the susceptibility of immunosuppressed cancer patients to infection, such as neutropenia during chemotherapy, altered gut flora because of frequent antibiotic administration, and disruption of skin and damage of epithelial surfaces of the tissues by cytotoxic chemotherapeutic agents [3, 4].

Bacteriuria is the presence of microbial pathogens in the urethra, bladder, ureter, and pelvis of the kidney [5, 6]. *Escherichia coli*, *Klebsiella pneumonia*, *Proteus mirabilis*, *Pseudomonas aeruginosa, Staphylococcus saprophyticus*, *Enterococcus faecalis,* and *Streptococcus agalactiae* are the leading cause of urinary tract infections [7, 8]. Obstruction of the urinary tract, use of a catheter, immunocompromised condition, estrogen deficiency, genetic predisposition, and sexual intercourse are common risk factors for urinary tract infection [9, 10]. Cancer patients are at high risk of bacterial infections due to the chemotherapy for cancer patients leads to severe and prolonged immunosuppression [11, 12]. The development of infections caused by multidrug-resistant bacteria has become a major health problem worldwide [13–15].

Urinary tract infection is a serious health problem in the community and health care settings. Cancer patients are at high risk of urinary infections due to cancer chemotherapy leads to severe and prolonged immunosuppression such as neutropenia during chemotherapy, altered gut flora because of frequent antibiotic administration, and disruption of skin and damage of epithelial surfaces of the tissues by cytotoxic chemotherapeutic agents [16, 17]. Due to this, it is a serious health problem that involves bacterial invasion and multiplication in the organs of the urinary tract system, and it is a usual problem for patients to visit outpatient departments [18–20]. Despite the widespread availability of antibiotics, urinary tract infection remains a worldwide therapeutic problem [21–24]. In Ethiopia, no study had been carried out before in this area. Hence, it was important to assess asymptomatic bacteriuria and antibiotic susceptibility patterns of uropathogens among cancer patients and apparently healthy blood donors at the University of Gondar comprehensive specialized referral hospital.

## 2. Methods

### 2.1. Study Area, Study Design, and Population

The study was conducted at the University of Gondar comprehensive specialized referral hospital. Gondar town has 8 health centers, 21 private clinics, and one referral hospital with a projected population of 323,900. The hospital serves for more than five million people of Gondar town and its surroundings. The hospital has different departments and 500 beds for admitted patients. A hospital-based comparative cross-sectional study was conducted to assess the prevalence of asymptomatic bacteriuria and antibiotic susceptibility patterns of bacterial isolates among cancer patients and apparently healthy blood donors at the University of Gondar comprehensive specialized referral hospital, Northwest Ethiopia, from January to June 2019. Cancer confirmed patients and apparently healthy blood donors were the study population. However, study participants who were unable to give sociodemographic information currently on antibiotic treatment and had a recent history of antibiotic treatment for the last three weeks at the time of data collection were excluded.

### 2.2. Ethical Approval

Ethical approval was obtained from the University of Gondar ethical review committee. Written legal permission was obtained from medical directors of the University of Gondar compressive specialized hospital. The objectives of the study were explained to the hospital directors, health-care providers, and patients; clarification also was given for patients before starting data collection. To keep confidentiality of information from participants, no personal identifiers were recorded in the client information extraction predesigned form and data secured from participant records were not available to anyone except for the main investigator.

### 2.3. Sample Size and Sampling Technique

A total of 120 cancer patients and 120 apparently healthy blood donors were enrolled using simple random sampling technique, and we took a 1 : 1 ratio of cancer patients and apparently healthy blood donors.

### 2.4. Sociodemographic Data and Urine Specimen Collection

A pretested questionnaire based on postulated risk factors was developed and modified to explore the objectives of the study. Then, sociodemographic characteristics and other relevant information were collected. Urine specimens were collected by a laboratory technologist by instructing the patients to collect approximately 10 ml to 15 ml midstream urine in clean, leak-proof sterile plastic cups at the University of Gondar compressive specialized referral hospital reception. Then, midstream urine samples were collected from each study participants after obtaining written informed consent and assent from the families of children and then transported to the Medical Microbiology Laboratory immediately.

### 2.5. Laboratory Identification Procedures

Each urine samples were inoculated onto a Cysteine-Lactose-Electrolyte Deficient agar (CLED) (Oxoid Ltd., England) by using a calibrated, sterile, nonreusable plastic loop 1 *μ*l (0.001 ml) and incubated aerobically at 37°C for 18 to 24 hours to check the growth, and urine cultures were considered as significant bacteriuria when colony forming units (CFUs) were ≥10^5^/ml of voided urine, and a single colony was picked and suspended in nutrient broth and then subcultured onto blood agar plate and MacConkey agar plate, finally incubated at 37°C for 24 hours for further identification. Bacterial identification was then done using standardized biochemical tests, namely, indole production, lactose fermentation, hydrolysis of urea, citrate utilization, lysine decarboxylation, and motility test for Gram-negative bacteria and for Gram-positive bacteria, mannitol fermentation, and catalase and coagulase tests.

### 2.6. Antimicrobial Susceptibility Testing

A suspension of a pure colony from each confirmed culture isolate was performed by using 0.85% sterile normal saline, and the suspension was adjusted at 0.5% MacFarland standard. Using a sterile cotton applicator stick, the suspension was distributed evenly on Muller-Hinton agar. Modified Kirby-Bauer disk diffusion technique was implemented for antibiotic susceptibility pattern using different antibiotics such as ampicillin (10 *μ*g), amoxicillin/clavulanate (30 *μ*g), ceftazidime (30 *μ*g), tobramycin (10 *μ*g), cefoxitin (30 *μ*g), vancomycin (30 *μ*g), tetracycline (30 *μ*g), penicillin (10 *μ*g), ciprofloxacin (5 *μ*g), norfloxacin (10 *μ*g), nitrofurantoin (300 *μ*g), nalidixic acid (30 *μ*g), and rifampicin (5 *μ*g). Then, we applied those antibiotics on Mueller-Hinton agar plate and incubated for 18–24 hours at 37°C. The zones of inhibition were measured, recorded, and interpreted as sensitive, intermediate, and resistant using the CLSI 2018 performance standards for antimicrobial susceptibility testing interpretation table. MDR isolates are bacterial strains which are nonsusceptible to greater than or equal to one antimicrobial agent in three or more antimicrobial categories [25].

### 2.7. Data and Laboratory Quality Control

The questionnaire was pretested before the actual study begins to make sure whether the questionnaire was appropriate and understandable. The collected data were checked daily for consistency and accuracy. Investigators were also following standard data collection process. Five percent (5%) of the prepared culture media were randomly selected and incubated aerobically for 24 hours at 37°C to cheek the sterility of the prepared culture media, and also known strains of *Staphylococcus aureus* (ATCC 25923) and *Escherichia coli* (ATCC 25922) were inoculated onto the prepared culture media to check the performance of the prepared culture media and antibiotic susceptibility test. Laboratory identification procedures such as inoculation of culture media, colony characterization, and measuring of antibiotic susceptibility testing were checked. Reagents for Gram stain and biochemical tests were checked using *Staphylococcus aureus* (ATCC 25923) and *Escherichia coli* (ATCC 25922).

### 2.8. Data Entry and Analysis

Data were entered to EPI Info version 7 to check data completeness and data clearance and then transferred to SPSS version-20 for analysis. The characteristics of the study populations were summarized using frequencies, mean, and standard deviation. Binary logistic regression was used to determine the strength of the association between variables. Moreover, adjusted odds ratio was computed using multivariate logistic regression for variables with *p* value ≤0.2 to control confounding variables. *p* value ≤0.05 was considered statistically significant at 95% CI.

## 3. Results

### 3.1. Sociodemographic Characteristics

In this study, a total of 240 study participants were included. Of these, 50% (120/240) were cancer patients and 50% (120/240) were apparently healthy blood donors. Among cancer patients, 58.3% (70/120) were females and 41.7% (50/120) were males, and also among apparently healthy blood donors, 68.3% (82/120) were males and 31.7% (38/120) were females. The majority, 61.7% (74/120) of cancer confirmed patients were from an urban resident, while the rest, 67.5% (81/120) apparently healthy blood donors were from a rural resident. Most of the cancer confirmed patients, 32 (26.7%), had breast cancer followed by colon cancer, 30 (25%). The mean age of the study subjects was 42 years with a range of 3–80 years. 35% (42/120) of cancer patients belonged to 41–60 years of age while 38.3% (46/120) of apparently healthy blood donors belong to 21–30 years of age (Table 1).

### 3.2. Prevalence of Asymptomatic Bacteriuria

The overall prevalence of asymptomatic bacteriuria in cancer patients attending the University of Gondar comprehensive specialized hospital was 23.3% (28/120) while the prevalence of asymptomatic bacteriuria in apparently healthy blood donors was 6.7% (8/120). Of the 28 bacterial isolates in cancer patients, 35.7% (10/28) of them were isolated from male participants while 64.3% (18/28) of them from female participants. Moreover, of the 8 bacterial isolates in apparently healthy blood donors, 50% (4/8) of isolates were from females and 50% (4/120) of them were isolated from male participants.

**Figure 1:**
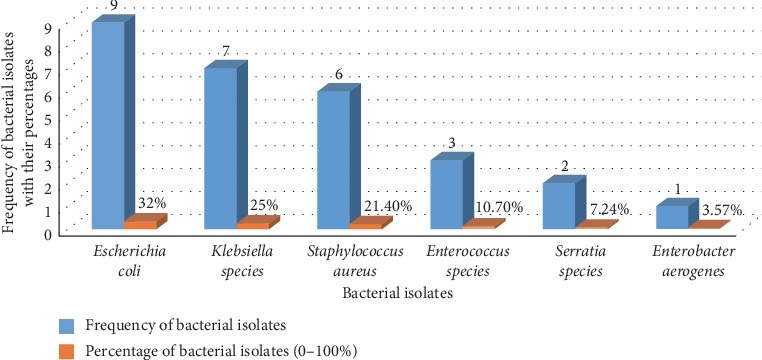
Frequency of bacterial isolates among cancer patients at the University of Gondar comprehensive specialized hospital, 2019.

**Figure 2:**
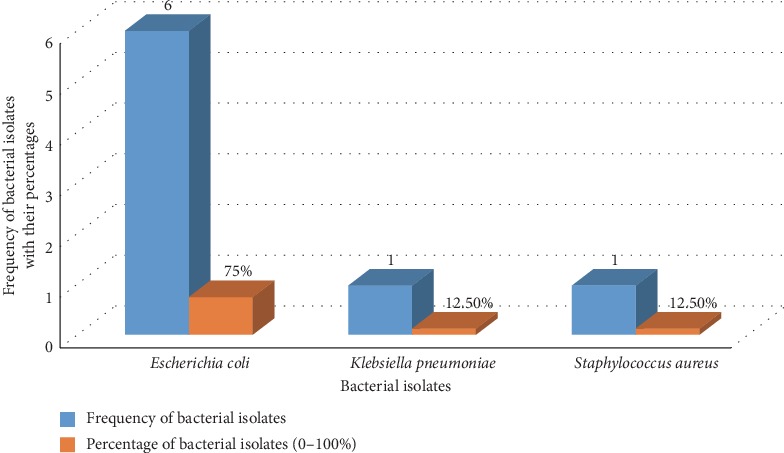
Frequency of bacterial isolates among apparently healthy blood donors at the University of Gondar comprehensive specialized hospital, 2019.

The predominant bacterial isolate in cancer patients was *E. coli* (32%), *Klebsiella* species (25%), *S*. *aureus* (21.4%), *Enterococcus* species (10.7%), *Serratia* species (7.14%), and *Enterobacter aerogenes* (3.57%) (Figure 1). In apparently healthy blood donors, *E*. *coli* (75%), *K. pneumonia* (12.5%), and *S*. *aureus* (12.5%) were the most frequently isolated bacteria (Figure 2). *E*. *coli* was the frequently isolated bacteria in both cancer patients and apparently healthy blood donors, 32% (9/28) and 75% (6/8), respectively. The prevalence of asymptomatic bacteriuria in females was 15% (18/120) in cancer patients while 3.3% (4/120) in apparently healthy blood donors. High prevalence of asymptomatic bacteriuria was observed in females as compared to males. From a total of cancer patients with asymptomatic bacteriuria, 32.1% (9/120) were in the age group of 40–60 years while 25% (7/120) were in the age group of above 60 years (Table 2).

### 3.3. Associated Risk Factors for Asymptomatic Bacteriuria

Sociodemographic data such as age, sex, residence, occupation, educational status, marital status, and monthly income of the patients had been analyzed to assess their contribution for asymptomatic bacteriuria. Similarly, history of hospitalization, history of surgery, history of catheterization, history of UTIs, type of cancer, and duration of cancer chemotherapy (follow-up time) had also been assessed for the association. Among the risk factors assessed in cancer patients, there were no statistically significant risk factors for asymptomatic bacteriuria (*P* > 0.05). However, MDR isolates are associated with a history of antimicrobial usage.

### 3.4. Antimicrobial Susceptibility Patterns of Bacterial Isolates

Bacterial antimicrobial susceptibility tests were performed for bacterial isolates; the antimicrobial susceptibility test result showed that majority of the isolates were sensitive for tobramycin (88.8% for *Escherichia coli,* 57.1% for *Klebsiella* species, 100% for *Serratia* species, and 100% for *Enterobacter aerogenes*), ciprofloxacin (77% for *Escherichia coli*, 71.4% for *Klebsiella* species, 100% for *Enterobacter aerogenes,* and 66.6% for *Staphylococcus aureus*), nitrofurantoin (77.8% for *Escherichia coli,* 85.7% for *Klebsiella* species, and 100% for *Serratia* species, *Enterobacter aerogenes,* and *Staphylococcus aureus*) and cefoxitin (100% for *E*. *coli*, 71.4% for *Klebsiella* species, and 100% for *E*. *aerogenes*) which were found to be efficient antibiotics (Table 3). However, most of the isolates were resistant to tetracycline (77.8% for *E*. *coli,* 42.9% for *Klebsiella* species, 66.7% for *Enterococcus* species, and 100% for *E*. *aerogenes* and *Serratia* species). *S*. *aureus* isolates were 100% susceptible to nitrofurantoin.

### 3.5. Prevalence of Multidrug-Resistant Isolates

Among the isolates, 46.4% (13/28) showed multidrug-resistance pattern in cancer patients, while in apparently healthy blood donors, 25% (2/8) showed multidrug-resistance pattern. The proportion of multidrug-resistant isolates among cancer patients for *E*. *coli* was 44.4% (4/9), *Klebsiella species* 57.1% (4/7), *S. aureus* 16.7% (1/6), *Enterococcus species* 100% (3/3), and *Serratia species* 50% (1/2) and among apparently healthy blood donors, *E. coli was* 16.7% (1/6) and *S*. *aureus* 100% (1/1).

## 4. Discussion

This study was carried out to assess the burden of asymptomatic bacteriuria and associated risk factors which could be the possible causes for bacteriuria in cancer patients attending at the University of Gondar comprehensive specialized hospital. In this study, the overall prevalence of asymptomatic bacteriuria in cancer confirmed patients was 23.3%. This finding of bacteriuria was in line with the reports in Korea (23.5%) [26] and India (20.1%) [27]. However, a higher prevalence of bacteriuria was reported in this study than the studies conducted in Texas (15%), Sweden (5%), and Japan (15%) [28–30]. On the other hand, this finding showed a lower prevalence of bacteriuria than those reported in Saudi Arabia (35.8%), India (34.7%, 32%), and Sudan (31.6%) [31–34]. This variation of bacteriuria from other studies might be due to the difference in the characteristics of the study population, quality of sampling, culturing techniques, geographical distribution, and diagnostic techniques.

The frequency of Gram-negative bacteria isolated from cancer patients in Egypt was 17.2% [35] from a urine sample, which is higher than our study. However, in our study (15.83%), Gram-negative bacteria were found from the urine of cancer patients, which is similar to the frequency of bacteriuria (15%) reported in a Japanese study [36]. Among Gram-negative isolates, *Escherichia coli* and *Klebsiella* species were isolated from 32.1% and 25.0%, respectively. In this finding, *E*. *coli* was the predominant isolate which is compatible with a study conducted in India (*E*. *coli* (40%) and *K. pneumonia* (25%)) [33], another study in India (*E*. *coli* (38.1%)) [34], and in Sudan (*E*. *coli* (39.2%) and *Klebsiella pneumonia* (19%)) [37]. Among Gram positives*, S*. *aureus* was the predominantly isolated bacteria followed by *Enterococcus* species, which is in line with a study conducted in Tamil Nadu, India [32], and in Nigeria [38]. This variation might be due to sample size variation, and we had included all types of cancer patients in our study, but other studies include specific cases of cancer patients.

The prevalence of asymptomatic bacteriuria among cancer patients (23.3%) was higher than that of apparently healthy blood donors (6.7%). This higher prevalence of asymptomatic bacteriuria in cancer patients might be due to the immunocompromised state of cancer patients by cancer chemotherapeutic agents. Moreover, in cancer patients, the prevalence of asymptomatic bacteriuria in females (64.3%) was higher than that of males (35.7%). The higher prevalence of asymptomatic bacteriuria in females might be due to women's urethra is short and located near the anus that allows relatively easy passage of bacteria into the bladder and urethral opening.

Many factors were assessed as risk factors for bacteriuria in cancer patients. However, no evidence was found to support the association between asymptomatic bacteriuria and sex, age, and cancer types (*P* > 0.05). Correspondingly, a previous study by Fukushima et al. showed that neither of the above factors mentioned in our study had no effect on the occurrence of bacteriuria [36]. On the contrary, bladder cancer had shown statistically significant association with urinary tract infection in a study done by Richards et al. [39]. A study by Fan et al. in 2017 had also identified an association between prostate cancer and bacteriuria [40]. Another study by Sun et al. in 2013 had also confirmed the association among urinary tract cancers and asymptomatic bacteriuria [8]. This difference might be due to the variation in study design and characteristics of the study population. In our study, catheterization and previous surgery and other independent variables were not significantly associated with bacteriuria. Consistently, surgery had been found to be less important in a study by Kim et al. in patients with bladder cancer [41].

In our study, the effect of ampicillin, augmentin, tetracycline, and penicillin were minimal while cefoxitin, nitrofurantoin, norfloxacin, nalidixic acid, and ciprofloxacin were found to be the most efficient antibiotics for bacterial isolates from both cancer patients and apparently healthy blood donors. Among cancer patients, *Enterococcus* species were resistant to five antimicrobials, one *Serratia* species isolate showed resistance to four antimicrobials, and three *klebsiella* species were resistant to three antimicrobials. Among the apparently healthy blood donors, one bacterial isolate of *S*. *aureus* showed multidrug resistance to three antimicrobials and one *E*. *coli* isolate was also resistant to three antimicrobials. The differences in susceptibility pattern of the isolates might be due to the differences in the management of antibiotics and geographical area. MDR isolates are associated with a history of antimicrobials; this might be due to the development of specific mechanisms of resistance through time.

## 5. Conclusion

In this study, the prevalence of asymptomatic bacteriuria among cancer patients (23.3%) was found to be higher than that of apparently healthy blood donors (6.7%). *Escherichia coli* was the most frequent isolate in both cancer patients and apparently healthy blood donors. *S*. *aureus* was the most commonly isolated Gram-positive bacteria in cancer patients. Cefoxitin, nitrofurantoin, norfloxacin, nalidixic acid, and ciprofloxacin were found to be the most efficient antibiotics. Based on the study findings, if asymptomatic bacteriuria is suspected and laboratory tests are not available, it is recommended that nitrofurantoin and ciprofloxacin should be used in preference to penicillin, augmentin, and tetracycline in the study area. Ministry of health should think about ampicillin, augmentin, and penicillin since they are inefficient to isolates.

## Figures and Tables

**Table 1:** Frequency of sociodemographic characteristics of cancer patients and apparently healthy blood donors at the University of Gondar comprehensive specialized hospital, Gondar, Northwest Ethiopia, 2019.

Variables		Cancer patients, *n* = 120 (%)	Blood donors, *n* = 120 (%)
Sex	Male	50 (41.7)	82 (68.3)
	Female	70 (58.3)	38 (31.7)

Age category in years	<10	8 (6.7)	0 (0)
	11–20	9 (7.5)	11 (9.2)
	21–30	15 (12.5)	46 (38.3)
	31–40	28 (23.3)	39 (32.5)
	41–60	42 (35)	20 (16.7)
	61–70	11 (9.2)	4 (3.3)
	>70	7 (5.8)	0 (0)

Marital status	Married	91 (78.8)	82 (68.0)
	Unmarried	27 (22.5)	38 (31.7)
	Divorced	1 (0.8)	0 (0)
	Widowed	1 (0.8)	0 (0)

Residences	Urban	74 (61.7)	81 (67.5)
	Rural	46 (38.3)	39 (32.5)

Educational status	Illiterate	50 (41.7)	19 (15.8)
	Read and write	9 (41.7)	8 (6.7)
	Elementary	20 (16.7)	9 (7.5)
	Secondary	21 (17.5)	23 (19.2)
	Twelve and above	20 (16.7)	61 (50.8)

Occupation	Governmental	22 (18.3)	60 (50)
	Nongovernmental	1 (0.8)	0 (0)
	Farmer	43 (35.8)	30 (25)
	Merchant	22 (18.3)	2 (1.7)
	Student	15 (12.5)	20 (16.7)
	Unemployed	17 (14.2)	0 (0)

Monthly income	<17 dollar	53 (44.2)	29 (24.2)
	18–33 dollar	27 (22.5)	16 (13.3)
	34–50 dollar	12 (10)	10 (8.3)
	51–67 dollar	6 (5.0)	8 (6.7)
	>68 dollar	22 (18.3)	57 (47.5)

**Table 2:** Independent variables examined for relations to urinary tract infections on cancer patients at the University of Gondar comprehensive specialized hospital, Gondar, Northwest Ethiopia, 2019.

Characteristics		Number (%)	Urinary tract infection					
			No. (%) +ve for UTI	No. (%) −ve for UTI	Crude OR (95% CI)	*P* value	Adjusted OR (95% CI)	*P* value
Sex	Female	70 (53.8)	18 (64.3)	52 (56.5)	1.4 (0.58–3.74)	0.47	—	—
	Male	50 (41.7)	10 (35.7)	40 (43.5)	1 (ref)	1 (ref)	—	

Residences	Urban	74 (61.7)	17 (60.7)	57 (62)	0.95 (0.40–2.26)	0.91	—	—
	Rural	46 (38.3)	11 (39.3)	35 (38)	1 (ref)	1 (ref)	—	

Age in years	<20 years	17 (14.3)	4 (14.3)	13 (14.1)	2.07 (0.48–8.97)	0.52	—	—
	21–30 years	15 (12.5)	2 (7.1)	13 (14.1)	4.14 (0.71–24.12)	0.33	—	—
	31–40 years	28 (21.4)	6 (21.4)	22 (23.9)	2.33 (0.63–8.64)	0.12	0.30 (0.156–4.46)	0.18
	41–60 years	42 (35)	9 (32.1)	33 (35.9)	0.41 (0.18–0.9)	0.20	0.25 (0.529–19.47)	0.09
	>60 years	18 (15)	7 (25)	11 (12)	1 (ref)	1 (ref)	1 (ref)	1 (ref)

Educational status	Illiterate	50 (41.7)	13 (46.4)	37 (40.2)	0.71 (0.20–2.5)	0.59	—	—
	Read and write	9 (7.5)	4 (14.3)	5 (5.4)	0.3 (0.05–1.7)	0.18	0.24 (1.05–124.54)	0.16
	Primary	20 (16.7)	2 (7.1)	18 (19.6)	2.3 (0.36–13.9)	0.38	—	—
	Secondary	21 (17.5)	5 (17.9)	16 (17.4)	0.8 (0.18–3.54)	0.77	—	—
	12 and above	20 (16.7)	4 (14.3)	16 (17.4)	1 (ref)	1 (ref)	1 (ref)	1 (ref)

Occupation	Farmer	43 (35.8)	12 (42.9)	31 (33.7)	0.25 (0.05–1.21)	0.08	0.53 (0.327–2.967)	0.089
	Merchant	22 (18.3)	7 (25)	15 (16.3)	0.20 (0.04–1.12)	0.07	4.14 (0.458–6.454)	0.13
	Students	15 (12.5)	4 (14.3)	11 (12)	0.26 (0.04–1.66)	0.26	—	—
	Unemployed	17 (14.2)	3 (10.7)	14 (15.2)	0.44 (0.07–3.00)	0.41	2.10 (0.177–1.646)	0.54
	Employed	23 (19.2)	2 (7.1)	20 (22.8)	1 (ref)	1 (ref)	1 (ref)	1 (ref)

Income	<17 dollar	53 (44.2)	15 (53.6)	38 (41.3)	0.25 (0.05–1.22)	0.08	8.64 (0.909–11.882)	0.12
	18–33 dollar	27 (22.5)	5 (17.9)	22 (23.9)	0.44 (0.08–2.53)	0.36	—	—
	34–50 dollar	12 (10)	3 (10.7)	9 (9.8)	0.30 (0.04–2.12)	0.23	—	—
	51–67 dollar	6 (5)	3 (10.7)	3 (3.3)	0.10 (0.01–0.0.87)	0.04	0.25 (0.542–15.58)	0.09
	>68 dollar	22 (18.3)	2 (7.1)	20 (21.7)	1 (ref)	1 (ref)	1 (ref)	1 (ref)

Marital status	Married	91 (75.8)	22 (78.6)	69 (75)	0.82 (0.30–2.23)	0.70	—	—
	Unmarried	29 (24.2)	6 (21.4)	23 (25)	1 (ref)	1 (ref)	1 (ref)	1 (ref)

History of hospitalization	Yes	104 (86.7)	23 (82.1)	81 (88)	0.63 (0.20–2.00)	0.42	—	—
	No	16 (13.3)	5 (17.9)	11 (12)	1 (ref)	1 (ref)	—	—

History of surgery	Yes	64 (53.3)	14 (50)	50 (54.3)	0.84 (0.36–2.00)	0.68	—	—
	No	56 (46.7)	14 (50)	42 (45.7)	1 (ref)	1 (ref)	—	—

History of catheterization	Yes	8 (6.7)	2 (7.1)	6 (6.5)	1.10 (0.21–5.80)	0.91	—	—
	No	112 (93.3)	26 (92.9)	86 (93.5)	1 (ref)	1 (ref)	—	—

History of previous UTI	Yes	4 (3.3)	2 (7.1)	2 (2.2)	0.29 (0.40–2.20)	0.22	—	—
	No	116 (96.7)	26 (92.9)	90 (97.8)	1 (ref)	1 (ref)	—	—

Type of cancer	Blood	12 (10)	1 (3.6)	11 (12)	0.92 (0.30–2.23)	0.38	—	—
	Colon	22 (18)	4 (14.3)	18 (19.6)	1.20 (0.21–5.80)	0.45	—	
	Bladder	9 (7.5)	2 (7.1)	7 (7.6)	0.41 (0.03–5.30)	0.38	—	
	Breast	32 (26.7)	9 (32.1)	21 (22.8)	0.30 (0.01–0.87)	0.17	0.47 (0.094–2.486)	0.49
	Lymph	22 (18.3)	7 (25)	15 (16.3)	0.44 (0.08–2.53)	0.15	0.70 (0.049–3.189)	0.64
	Thyroid	23 (19.2)	5 (17.9)	20 (21.7)	1 (ref)	1 (ref)	1 (ref)	1 (ref)

Cancer treatment follow-up	<1 year	97 (80.8)	25 (89.3)	72 (78.2)	0.26 (0.03–2.13)	0.2	3.32 (0.542–15.58)	0.33
	1–2 years	11 (9.2)	2 (7.1)	9 (9.8)	0.41 (0.03–5.30)	0.4	—	—
	>2 years	12 (10)	1 (3.6)	11 (12)	1 (ref)	1 (ref)	1 (ref)	1 (ref)

**Table 3:** Antibiotic susceptibility patterns of pathogenic bacterial isolates among cancer patients at the University of Gondar comprehensive specialized hospital, Gondar, Northwest Ethiopia, 2019.

Antibiotics	Cancer patients																	
	*E. coli* (9)			*Klebsiella* species (7)			*S. aureus* (6)			*Enterococcus* species (3)			*Serratia* species (2)			*E. aerogenes* (1)		
	*S* (%)	*I* (%)	*R* (%)	*S* (%)	*I* (%)	*R* (%)	*S* (%)	*I* (%)	*R* (%)	*S* (%)	*I* (%)	*R* (%)	*S* (%)	*I* (%)	*R* (%)	*S* (%)	*I* (%)	*R* (%)
Ampicillin	11.1	11.1	77.8	14.3	0	85.7	N/A	N/A	N/A	0	0	100	0	0	100	0	0	100
Amoxicillin/clavulanate	44.5	22.2	33.3	57.1	0	42.9	N/A	N/A	N/A	N/A	N/A	N/A	50	0	50	100	0	0
Ceftazidime	100	0	0	57.1	0	42.9	N/A	N/A	N/A	N/A	N/A	N/A	0	0	100	100	0	0
Tobramycin	88.8	0	11.2	57.1	0	42.9	N/A	N/A	N/A	N/A	N/A	N/A	100	0	0	100	0	0
Cefoxitin	100	0	0	71.4	0	28.9	83.3	18.7	0	N/A	N/A	N/A	50	0	50	100	0	0
Vancomycin	N/A	N/A	N/A	N/A	N/A	N/A	N/A	N/A	N/A	100	0	0	N/A	N/A	N/A	N/A	N/A	N/A
Tetracycline	22.2	0	77.8	42.9	14.2	42.9	33.3	33.3	33.3	33.3	0	66.7	0	0	100	0	0	100
Penicillin	N/A	N/A	N/A	N/A	N/A	N/A	16.7	0	83.3	0	0	100	N/A	N/A	N/A	N/A	N/A	N/A
Ciprofloxacin	77.8	0	22.2	71.4	0	28.9	66.6	16.7	16.7	66.7	0	33.3	50	0	50	100	0	0
Norfloxacin	77.8	0	22.2	71.4	0	28.9	83.3	0	18.7	0	33.3	66.7	50	0	50	100	0	0
Nitrofurantoin	77.8	0	22.2	85.7	0	14.3	100	0	0	100	0	0	100	0	0	100	0	0
Nalidixic acid	100	0	0	85.7	0	14.3	N/A	N/A	N/A	N/A	N/A	N/A	0	0	100	100	0	0
Rifampicin	N/A	N/A	N/A	N/A	N/A	N/A	N/A	N/A	N/A	33.3	0	66.7	N/A	N/A	N/A	N/A	N/A	N/A

*S* = susceptible; *I* = intermediate; *R* = resistance; N/A = not applicable.

## Data Availability

All data generated or analyzed during this study are included within this article. Data that support the findings of this study are also available from the corresponding author upon reasonable request.

## Abbreviations

ATCC: American Type Culture Collection
BAP: Blood agar plate
CLED: Cysteine-Lactose-Electrolyte Deficient
CLSI: Clinical and Laboratory Standards Institute
CFU: Colony forming unit
EPI: Epidemiological information
IARC: International Agency for Research on Cancer
MAC: MacConkey agar
MUH: Muller-Hinton agar
SPSS: Statistical package for the social sciences
UTI: Urinary tract infection
USD: United States dollar
WHO: World Health Organization.
